# Exploring the Motivations for Punishment: Framing and Country-Level Effects

**DOI:** 10.1371/journal.pone.0159769

**Published:** 2016-08-03

**Authors:** Jonathan E. Bone, Katherine McAuliffe, Nichola J. Raihani

**Affiliations:** 1 Department of Genetics, Evolution and Environment, University College London, Gower Street, London, United Kingdom, WC1E 6BT; 2 CoMPLEX, University College London, Gower Street, London, United Kingdom, WC1E 6BT; 3 Department of Psychology, Boston College, Chestnut Hill, Boston, MA, United States of America, 02467; 4 Department of Experimental Psychology, University College London, 26 Bedford Way, London, United Kingdom, WC1H 0AP; University of Portsmouth, UNITED KINGDOM

## Abstract

Identifying the motives underpinning punishment is crucial for understanding its evolved function. In principle, punishment of distributional inequality could be motivated by the desire to reciprocate losses ('revenge') or by the desire to reduce payoff asymmetries between the punisher and the target ('inequality aversion'). By separating these two possible motivations, recent work suggests that punishment is more likely to be motivated by disadvantageous inequality aversion than by a desire for revenge. Nevertheless, these findings have not consistently replicated across different studies. Here, we suggest that considering country of origin—previously overlooked as a possible source of variation in responses—is important for understanding when and why individuals punish one another. We conducted a two-player stealing game with punishment, using data from 2,400 subjects recruited from the USA and India. US-based subjects punished in response to losses *and* disadvantageous inequality, but seldom invested in antisocial punishment (defined here as punishment of non-stealing partners). India-based subjects, on the other hand, punished at higher levels than US-based subjects and, so long as they did not experience disadvantageous inequality, punished stealing and non-stealing partners indiscriminately. Nevertheless, as in the USA, when stealing resulted in disadvantageous inequality, India-based subjects punished stealing partners more than non-stealing partners. These results are consistent with the hypothesis that variation in punitive behavior varies across societies, and support the idea that punishment might sometimes function to improve relative status, rather than to enforce cooperation.

## Introduction

The factors underpinning decisions to cooperate and to punish others have traditionally been studied in stylized economic games in laboratories, mostly using Western undergraduates as the representative sample [1–4]. One traditional paradigm is the public goods game, where group members' contributions are costly in that they benefit the group at the expense of the contributor [3]. In such settings, many subjects are willing to forfeit a portion of their endowment to fine uncooperative players or cheats (defined in this context as individuals who violate distributive norms); a behavior that is interpreted as punishment [3]. Individuals that interact with cheating partners often experience negative emotions, and the strength of these emotions predicts investment in costly punishment [3, 5–8]. Nevertheless, these negative emotions could stem from two (not mutually exclusive) sources [9]. First, since cooperators that interact with cheats incur losses, punishment might reflect the desire to inflict reciprocal harm on a cheating partner (i.e. 'revenge'; [10]). Punishment that is motivated by revenge might conceivably function to deter cheating interaction partners from repeating their harmful actions again in subsequent interactions with the punisher [10]). Evidence from humans and non-human species suggests that punishment might often achieve this deterrent function [3,11,12] but see [13–16]). Nevertheless, in stereotypical laboratory games, cheats typically also end up with higher payoffs than cooperators meaning that, in addition to losses, victims also experience disadvantageous inequality. This raises an alternative possibility: that punishment is motivated by disadvantageous inequality aversion (the willingness to give up material payoffs to avoid receiving a lower payoff than others [17]) [9]. Punitive sentiments that are motivated by disadvantageous inequality aversion (rather than a desire for revenge) are arguably more consistent with the idea of a fitness-leveling, rather than a deterrent, function of punishment [18].

Empirical studies that have disentangled whether subjects experience losses or inequality suggest that punishment might be motivated more by inequality aversion than by the desire for revenge (e.g. [2,19, 20]). For example, using a random income game (where no cheating occurred and the desire to inflict reciprocal harm could therefore be ruled out), Dawes et al. [2] showed that subjects would pay to reduce the income of the richest players in the group (and also to increase the earnings of the poorer members). A follow-on study showed that the preference for equal outcomes in the random income game was significantly associated with willingness to punish cheats in a public goods game, suggesting that egalitarian motives underpin the desire to punish cheats in social interactions [20].

Raihani & McAuliffe ([19] hereafter R&M) provided a more direct empirical test of the relative importance of losses versus inequality in predicting punishment. They used a two-player game, where player one (P1) was given an endowment and player two (P2) was allowed to steal some of this endowment, if they wished. Importantly, P1 experienced the same losses if P2 decided to steal but different relative outcomes, according to one of three treatments. Specifically, after P2 stealing, P1 either remained better off than P2 (advantageous inequality); had the same payoff as P2 (equal outcomes); or was worse off than P2 (disadvantageous inequality). P1 was then allowed to pay a fee to punish P2. This design allowed the possibly separate effects of incurring losses versus experiencing disadvantageous inequality on P1's decision to punish P2 to be separated. Notably, P1 only punished a stealing P2 more than a non-stealing P2 in the condition where P2 stealing resulted in disadvantageous inequality for P1, thereby supporting the idea that punishment decisions are motivated primarily by disadvantageous inequality rather than by a desire for revenge *per se* [19].

In an attempt to replicate and extend R&M's study, Bone & Raihani ([21], hereafter B&R) used a similar game structure. As in R&M, B&R showed that players were more likely to punish stealing partners if stealing resulted in disadvantageous inequality rather than equality or advantageous inequality. However, unlike R&M, B&R found that P1 was more likely to punish a stealing P2 than a non-stealing P2, even when stealing did not result in disadvantageous inequality. Similarly, P1 often punished P2 even when offered a punishment option that could not reduce inequality between them both (see [22] for similar results). [21] These findings suggest that punishment stems from both an aversion to inequality *and* a desire for revenge, and therefore seem to contradict the previous results. The discrepancy between these different patterns of results raises an important question: could it be that punishment of distributional inequality is driven by inequality aversion in some contexts and losses in others? We designed the current study to tease apart possible explanations for the differences between the findings of the original R&M study and the more recent B&R study. In doing so, we hoped to shed light on the contexts in which motives based on revenge or inequality aversion, respectively, are a more important driver of the decision to punish.

Our initial hypothesis was that methodological details could explain the discrepancy in the results of the two studies. In R&M [19], subjects were initially endowed with $0.70 which, if they were paired with a stealing partner, was reduced to $0.50. In contrast, in B&R [21], subjects started out with $1.10, which would be reduced to $0.90 if the partner stole $0.20. Although the losses experienced were consistent across the two studies ($0.20 in both), it is possible that the loss was perceived as greater in B&R due to a phenomenon called the left-digit effect [23], where losses that result in the left digit changing (i.e. from $1.10 to $0.90) are perceived as larger than those where the left digit does not change (i.e. as in $0.70 to $0.50). Our second hypothesis was that variation in the cost of punishing a stealing partner (relative to the punisher's endowment) was responsible for the different findings. Although the same fee to fine ratio (1:3) was used in both studies, punishment in R&M cost twice as much as in B&R ($0.10 versus $0.05, respectively) meaning that, in B&R, punishers both had a higher endowment to spend on punishment and could punish a partner more cheaply. People are sensitive to variation in the cost of punishing and invest more in punishment when it is cheaper [10, 24]. The current study was therefore designed to control for these confounding methodological details.

Our third hypothesis was that variation in punishment across R&M and B&R was due to societal differences between players. Data for both previous studies were collected via the online crowdsourcing platform, Amazon Mechanical Turk (MTurk; www.mturk.com), where the vast majority of workers hail from either the USA or India [25]. While R&M [19] recruited participants from both the USA and India, B&R [21] restricted participation to subjects based in the USA. Importantly, R&M did not control for possible country-level differences in subjects' behavior. If there are systematic differences in the way that India-based versus US-based subjects behave in economic games (e.g. see [26]), particularly with respect to punishment, then the different demographic sampling could explain the different results we saw across the two studies. Previous work has shown stark cross-cultural differences in the propensity to punish, both when punishment is aimed at social cheats [27–29], and when punishment is aimed at non-cheating or overtly cooperative individuals ('antisocial punishment'; [29, 30]. Uncovering differences in propensity to punish under different conditions between the US-based and India-based players on MTurk might therefore help us explain the discrepancies in the results of the previous studies and, more generally, to gain insights into country-level effects on the motives underpinning punishment decisions.

## Materials and Methods

### Experimental Protocol

This project was approved by the University College London ethics board under the project number 3720/001. Prior to taking part in the study, subjects were required to tick a box to indicate that they understood that they were taking part in scientific research and that their participation was voluntary. No deception was used in this study and participants were not debriefed as to the purpose of the study after the game. All data were collected in November 2014 using Amazon Mechanical Turk (see S2 Appendix for details and justification for online data collection using MTurk). Using MTurk allowed us to recruit a more diverse demographic sample than the typical western, educated, industrialized, rich and democratic (WEIRD, [4]) samples used in the majority of behavioral experiments [31]. Werecruited 2,400 subjects from 24 countries (S1 Table), with the vast majority (95%) hailing from USA (n = 1941) or India (n = 315).

Subjects played a modified version of the game used in R&M [19] and B&R [21] (described above and see S1 Appendix for game instructions). Participation in this experiment was restricted to subjects who had previously participated in at least one MTurk task and who had an approval rating of at least 75% for their prior MTurk work. MTurk workers are identified by a unique 14-digit worker ID rather than their names [32]. Subjects were told that their ID would not be revealed to their partner in the game, thus ensuring anonymity. Of the 2,400 subjects recruited to play the game, 1,200 were randomly assigned to the role of 'player 1' (P1) and the remaining 1,200 to the role of 'player 2' (P2). To be eligible to participate in the study, all subjects had to answer correctly three comprehension questions about the game. Prior to taking part, subjects also provided demographic information on their age, gender, education, income levels and country of origin (S2 Table).

The game consisted of two stages as follows:

**Stage 1:** Subjects were informed of their own initial endowment and the endowment of the partner; and P2 was then given the choice to take $0.20 of P1's bonus or to do nothing (as in R&M, [19]).
**Stage 2:** P1 was given the option to punish P2 (framed as 'reducing P2's bonus' in the game instructions; S1 Appendix), by paying $0.10 to reduce P2's bonus by $0.30 (as in R&M, [19]).

This experiment had a 3 x 4 design. Each player was allocated to one of three treatments (which determined P1's initial endowment, Table 1) and, within each treatment, there were four different scenarios (which determined P2's initial endowment, Table 1). Players were matched with partner’s ex-post (as in [19, 19, 21, 26]).

**Table 1:** Treatments, scenarios and respective outcomes if P2 stole from P1.

Treatment	P1's endowment	Scenario	P2's endowment	Outcome if P2 stole (P1-P2)	Sample size (P2 stole)	Sample size (P2 did not steal)
1. Replication	$0.70	A: Advantaged	$0.10	$0.50–$0.30	49	50
		B: Equal	$0.30	$0.50–$0.50	50	50
		C: Disadvantaged	$0.50	$0.50–$0.70	50	50
		D: Equality ruined	$0.70	$0.50–$0.90	50	50
2. Left-digit effect	$1.10	A: Advantaged	$0.50	$0.90–$0.70	49	50
		B: Equal	$0.70	$0.90–$0.90	50	50
		C: Disadvantaged	$0.90	$0.90–$1.10	49	50
		D: Equality ruined	$0.90	$0.90–$1.30	50	50
3. Relative punishment cost	$1.30	A: Advantaged	$0.70	$1.10–$0.90	50	50
		B: Equal	$0.90	$1.10–$1.10	50	50
		C: Disadvantaged	$1.10	$1.10–$1.30	50	50
		D: Equality ruined	$1.30	$1.10–$1.50	50	49

Initial endowments allocated to P1 and P2 in each treatment and scenario, and the corresponding the outcome if P2 stole. 'Advantaged' means that P1 remained better off than a stealing P2; 'Equal' means that P1 and P2 had equal payoffs after P2 stole; 'Disadvantaged' refers to the scenario where P2 was initially worse off than P1 but, by stealing, rendered P1 worse off; and 'Equality ruined' means that endowments were initially equal, but P2 stealing rendered P1 worse off. Sample sizes of P1’s who interacted with a stealing / non-stealing P2 according to the treatment and scenario are given.

P1's initial endowment varied according to the experimental treatment (see Table 1). For a direct replication of R&M [19], P1 started with $0.70 (Treatment 1). To test whether the left-digit effect [23] could explain variation in punishment, P1 started with $1.10 (Treatment 2). To test whether investment in punishment would be higher when the cost of punishment was a relatively smaller proportion of the initial endowment, P1 started with $1.30 (Treatment 3). P2's initial endowment varied according to the scenarios (A-D) outlined in Table 1. Losses incurred by P1 if P2 stole were constant across all scenarios and treatments ($0.20). Nevertheless, the outcome inequality varied across the four scenarios. In scenarios A–C, P1 started out better off than P2; but in scenario D, P1 and P2 started with an equal endowment. If P2 decided to steal from P1, the following outcomes ensued:

Scenario A: P1 remained better off than P2 ('advantageous inequality);
Scenario B: P1 and P2's final outcomes were equal ('equality');
Scenario C: P1 ended up worse off than P2 ('disadvantageous inequality');
Scenario D: P1 ended up worse off than P2 ('equality ruined disadvantageous inequality').

Thus, the four scenarios for each treatment allowed us to disentangle the possibly separate effects of experiencing losses versus experiencing disadvantageous inequality on P1's decision to punish P2. We included scenarios C and D to further delineate two potential motivations that might have driven P1 to punish a stealing P2. In Scenario C, P2 was initially worse off than P1 but could become better off by stealing from P1. In Scenario D, initial endowments of P1 and P2 were equal, but P2 stealing would tip the outcome in favor of P2 having more than P1. So, both scenarios represent disadvantageous inequality from P1's point of view, but in Scenario D there is the possibility to test whether any punishment can be attributed to P1's 'frustration' that the starting bonuses had initially been equal but were now tipped in favor of P2 (in other words, frustration that a fair outcome had been rejected by P2). Hereafter, for ease, we refer to scenario D as the 'equality ruined' condition.

### Analysis

First, we asked whether the left-digit effect or the relative cost of punishment affected P1's decision to punish a stealing P2. Under the left-digit hypothesis, P1 should be more likely to punish a stealing P2 in the ‘left-digit effect’ treatment (starting bonus $1.10; Table 1) relative to the ‘replication’ or ‘relative punishment cost’ treatments, even when stealing did not result in disadvantageous inequality for P1. Conversely, under the relative punishment cost hypothesis, P1 should be more likely to punish a stealing P2 in the 'relative punishment cost' treatment, relative to the 'replication' or 'left-digit effect' treatments. We explored these relationships using a chi-squared test. Data were restricted to instances where P2 stole from P1 and when stealing did not result in disadvantageous inequality for P1 (n = 297).

Next, we explored how country of origin and outcome inequality affected P1's punishment decisions. We used a generalized linear model (GLM), with the term 'punish' set as a binary response term (1 = P1 punished P2; 0 = P1 did not punish P2). As in R&M [19] and B&R [21], we were interested in how experiencing losses versus disadvantageous inequality affected P1's decision to punish P2. We therefore created a dummy explanatory term called 'outcome' that captured this variable. The term 'outcome' was a 3-level categorical variable with the levels ‘P2 didn’t steal' (P2 did not steal from P1); 'P2 stole no DI' (P2 stole but the stealing did not result in disadvantageous inequality); and 'P2 stole DI' (P2 stole and the outcome was disadvantageous inequality for P1). We included the following explanatory terms in the GLM: 'outcome'; 'equality ruined' (1 = P2 stealing resulted in a formerly equal outcome becoming unequal; 0 = otherwise); and, 'country' (a 2-level factor specifying P1's country of origin: India / USA). We also included the two-way interaction between country and outcome to assess whether players from India versus USA responded differently to different outcomes. Our preliminary chi-squared analysis (described above) showed that the starting bonus was not an important predictor of P1's punishment decision. We therefore combined data for all treatments (‘replication’, ‘left-digit effect’ and ‘relative punishment cost’) for this analysis. Since the vast majority (95%) of our subjects allocated to the P1 role were recruited from either the USA (n = 962) or from India (n = 176), we restricted data to responses from these subjects. The remaining 57 subjects allocated to the P1 role hailed from 24 different countries (S1 Table), rendering the sample size insufficient to draw meaningful inference about country-level effects on behavior for these individuals. We therefore had a sample size of 1138 punishment decisions for this model.

### Statistical Methods

Data were analyzed using R version 3.0.3 (www.r-project.org). All proportions are reported with 95% confidence intervals. For the GLM, we used an information-theoretic approach with model averaging, (as described in [33]), to determine the relative importance of the explanatory terms included in each model. Under an information-theoretic approach, a series of candidate models are generated, with each model representing a biological hypothesis. Rather than testing a null hypothesis, the relative degree of support for each model from the candidate set is calculated [34]. Initially, we specified a global model which included all the explanatory terms specified above. The input variables were centered by subtracting the mean [35], which allows averaging over models that include different interaction terms [33]. We used the package MuMIn [36] to derive and compare submodels from the initial global model. Models were compared to one another using Akaike's Information Criterion corrected for small sample sizes (AICc; [37]). A subset of 'top models' was defined by taking the best model (the model with the lowest AICc value) and any models within 2AICc units of the best model (following [34]). We computed the average parameter estimates for each term included in the top model set, as well as the relative importance of the term. Importance is calculated by summing the Akaike weights of all models where the term in question is included in the model. Akaike weights represent the probability of a given model being the true model (compared to other candidate models in the set) [34]. Importance can therefore be thought of as the probability that the term in question is a component of the best model [38]. In the results section, we only present the parameter estimates from the top models (those that were within 2 AICc units of the best model). All data and R code are available in S3 and S4 Appendices.

## Results

Neither the left-digit effect nor the relative cost of punishing seemed to affect P1's punishment decisions, in scenarios where stealing did not result in disadvantageous inequality (scenarios A and B) for P1. In treatment 1(starting bonus $0.70), P1 punished P2 on 12 / 99 (12.1%) occasions; in treatment 2 (starting bonus $1.10), P1 punished P2 on 16 / 99 (15.0%) occasions; and in treatment 3 (starting bonus $1.30), P1 punished P2 on 10 / 99 (10.2%) occasions. These differences across treatments were not significant at conventional levels (Chi-squared test: X = 1.69, df = 2, P = 0.43).

Our data did not support the idea that P1 punishment was motivated by frustration at P2 stealing turning previously equal outcomes into unequal ones. The term 'equality ruined' was a component of the top models, but the confidence intervals for this term spanned zero indicating that it was not an important driver of P1's punishment decision (Table 2).

**Table 2:** Explanatory terms included in the top models for the dependent variable "P1 punished P2".

Parameter	Estimate	Unconditional SE	Confidence Interval	Relative Importance
Intercept	-2.75	0.20	(-3.14, -2.36)	
Country (India / USA)	-1.93	0.30	(-2.52, -1.35)	1.00
Outcome				1.00
P2 stole no DI	2.10	0.42	(1.27, 2.93)	
P2 stole DI	3.31	0.41	(2.51, 4.12)	
Outcome x Country				1.00
P2 stole no DI	2.40	0.67	(1.09, 3.71)	
P2 stole DI	2.10	0.64	(0.85, 3.36)	
Equality ruined	-0.05	0.17	(-0.69, 0.34)	0.31

Estimates, unconditional standard errors, confidence intervals and relative importance for parameters included in the top models. Standard errors are unconditional, meaning that they incorporate model selection uncertainty. Outcome is a 3-level categorical variable: ‘P2 didn’t steal’ = player 2 did not steal; ‘P2 stole no DI’ = player 2 stole but this did not result in disadvantageous inequality for P1; and ‘P2 stole DI’ = player 2 stole and this resulted in in disadvantageous inequality for P1. For outcome, 'P2 didn’t steal' was the reference level. Estimates from the same model when the reference level for outcome is ' P2 stole no DI' presented in S3 Table).

**Fig 1:**
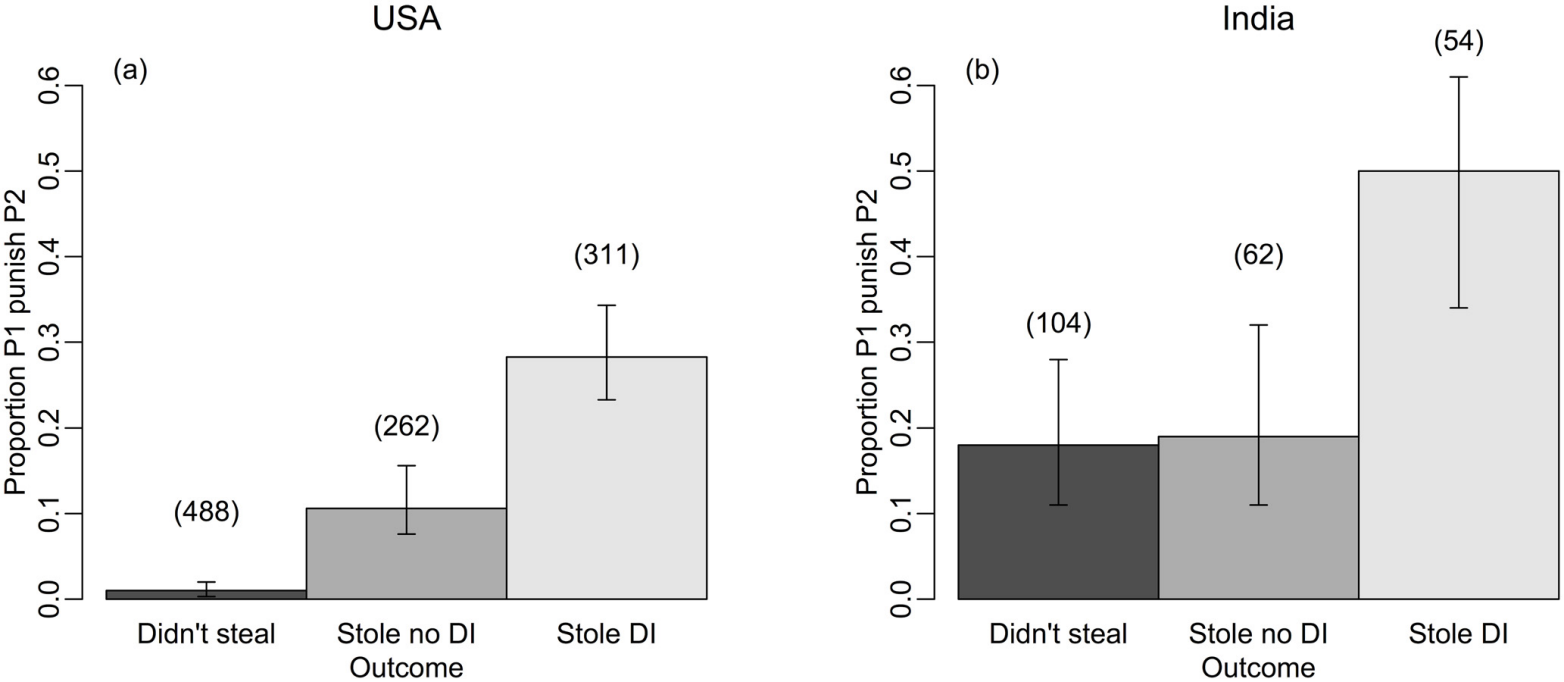
The proportion of P1 who punished when P2 didn’t steal (‘Didn’t steal’), P2 stole but the stealing did not result in disadvantageous inequality (‘Stole no DI’) or P2 stole and the outcome was disadvantageous inequality for P1 (‘Stole DI’). Data are shown for players based in **a)** the USA and **b)** India. Error bars show the 95% binomial confidence intervals (Agresti-Coull method). Sample sizes for each condition are indicated in parentheses. Plots are generated from raw data.

Instead, our data suggest that country of origin fundamentally affected punitive decisions. On average, India-based subjects were more punitive than US-based subjects (Table 2). In addition, subjects from India and the US responded differently to outcome (the variable describing whether the partner stole; and whether this resulted in disadvantageous inequality if so, Table 2; Fig 1). US-based subjects were more likely to punish stealing than non-stealing partners (even when stealing did not result in disadvantageous inequality, Fig 1a). Nevertheless, US-based subjects were also sensitive to inequality and punished stealing partners even more when stealing resulted in disadvantageous inequality (proportion punishing = 0.11 (0.08, 0.16) and 0.27 (0.23, 0.34), respectively; Fig 1a).

The patterns for India-based subjects were different. Subjects from India were more likely than US-based subjects to punish 'antisocially' (Fig 1). Moreover, so long as stealing did not produce disadvantageous inequality, India-based subjects punished stealing and non-stealing partners at equivalent levels. In other words, Indian subjects punished 'antisocially' at higher levels than US-based players and did not increase punishment in response to experiencing losses in the absence of disadvantageous inequality (Fig 1b). Nevertheless, when stealing *did* result in disadvantageous inequality (Scenarios C and D), India-based subjects were even more likely to punish their partner (proportion punishing = 0.19 (0.11, 0.32) and 0.50 (0.34, 0.66), respectively; Fig 1b). Thus, India-based subjects only punished stealing partners more than non-stealing partners when stealing resulted in disadvantageous inequality; whereas US-based subjects were sensitive to both losses and outcome inequality when making a punishment decision (Table 2). To check the robustness of these findings, we repeated these analyses using the data originally collected for R&M (see S5 Appendix). In R&M [19], 130 subjects allocated to P1 role were from India compared with 97 who were from the USA. Analyzing the variation in punishment revealed the same patterns as for the current dataset. India-based players were more likely to punish antisocially (proportion punishing = 0.16 (0.08, 0.28) compared with 0.03 (0.00, 0.12), S5 Appendix, S1 Fig) but were nevertheless still more likely to punish when stealing created disadvantageous inequality than when stealing did not result in disadvantageous inequality. Similarly, US-based players punished stealing partners more than non-stealing partners—but especially when stealing produced disadvantageous inequality (S5 Appendix, S1 Fig).

## Discussion

This study was designed to explore differences in revealed motivations for punishment in a two-player stealing game. Discrepancies in the results from previous studies do not seem to be explained by minor methodological differences (specifically, differences in stake size or the relative cost of punishment). Instead, we suggest that variation in the tendency to punish a stealing partner is largely explained by subjects' country of origin. For US-based subjects, the decision to punish was affected both by whether the partner stole and by whether the punisher experienced outcome inequality. Thus, the motives underpinning punishment apparently stemmed both from a desire for revenge and from the disutility associated with experiencing relatively lower payoffs than a partner (as in [21]). US-based subjects were very unlikely to punish a non-stealing partner. This pattern was not replicated for the Indian subjects, where 'antisocial' punishment was an order of magnitude higher. Indeed, so long as stealing did not result in disadvantageous inequality, India-based subjects punished stealing and non-stealing partners at statistically indistinguishable levels. By contrast, punishment was increased when stealing resulted in disadvantageous inequality for the punisher.

A non-negligible proportion of players were prepared to incur a cost to harm their partner, even when their partner did not steal any money (Fig 1). The tendency to pay to harm a non-stealing (or even an overtly cooperative) partner has been observed in several other studies (e.g. [2, 22, 27–30, 39–43], where it has variously been described with the labels 'antisocial punishment' or 'spite'. For ease, we will stick with the term 'antisocial punishment' to describe the punishment of a non-stealing partner in this paper. Although antisocial punishment was more common among the India-based than the US-based subjects, Indian subjects were generally more punitive than US-based players in all treatments and scenarios.

Bearing in mind that increased antisocial punishment may simply have been a by-product of being more punitive overall, we note that this finding mirrors previous studies that have shown cross-cultural variation in antisocial punishment [29,30]. Cross-cultural variation in antisocial punishment is thought to be predicted by weak rule of law, which is itself negatively linked to Gross Domestic Product (GDP) [30]. According to the World Bank [44], in 2013 the GDP of the USA was 16,800,000 million dollars, compared with 1,876,797 million dollars for India in the same year. This i supports previous evidence showing that GDP is negatively associated with citizens' propensity to punish antisocially. In addition, [30] showed that antisocial punishment was associated with low scores for civic norms of cooperation. Following [30] we used the questions from the most recent World Values Survey (2010–2014) [45] to calculate mean scores for civic norms of cooperation for Indian and US citizens (see S6 Appendix). We calculated a mean score of 8.73 for US citizens (comparable to the score of 8.65 as calculated by [30], using data from 1999–2004, S6 Appendix). For Indian citizens, we calculated a mean civic norms score of 6.84, which is lower than for any of the countries that were included in the Herrmann et al. [30] analysis. Thus, our results do seem to corroborate the idea that low civic norms of cooperation and GDP are associated with increased tendency for antisocial punishment.

In stereotypical public goods games, punishment is most often directed from cooperative individuals towards uncooperative targets. When targets are aware of who punished them and are given the chance to counter-punish, many individuals do choose this option [13,14, 16, 46–50]. Thus, antisocial punishment might sometimes reflect retaliation by free-riders who were punished by cooperative partners, or a pre-emptive strike against expected punishment in subsequent rounds [39,42]. In our study, however, retaliation (pre-emptive or otherwise) can be ruled out as a motive underpinning antisocial punishment since the punishers did not receive any previous punishment to retaliate against. We can also rule out the possibility that individuals used antisocial punishment strategically to deter partners from punishing in subsequent rounds, since this was a one-shot interaction. Instead, our findings lend more support to the recent idea that spiteful actions (including antisocial punishment) might simply reflect aggressive competition for status (as proposed by [39–43]. This idea is supported by previous studies, where the tendency to punish antisocially tends to reduce or even disappear when fee to fine ratios are adjusted such that punishers cannot improve their standing relative to that of the target [39].

Conceiving of punishment as an aggressive act, designed to improve relative status, can go some way to explaining cross-cultural differences in antisocial punishment use, since the benefits of acquiring higher status than others might vary with socio-ecological factors such as rule of law and resource availability (for which GDP might provide a reasonable proxy, [30]). For example, in a recent empirical study, Prediger et al. [43] used the 'joy of destruction' game [40] to show that willingness to engage in costly spiteful behavior correlates positively with resource scarcity. This empirical result supports earlier theoretical work which has shown that costly spite (aimed at competitors) can be favored under resource scarcity [51, 52]. If 'spiteful' or antisocial punishment functions to improve status (rather than to deter partners from cheating) it need not be motivated by a desire for revenge and might instead be aimed rather indiscriminately against stealing and non-stealing partners. This hypothesis would, nevertheless, predict increased punishment of stealing partners (relative to non-stealing partners) when stealing results in disadvantageous inequality. The empirical data from our India-based subjects precisely matches these predictions. Thus, our data support the idea that punishment might be used at least in some contexts to establish or maintain dominance over peers. An obvious future extension to this study would be to investigate whether we would observe similar punishment patterns when the fee to fine ratio is 1:1 (or lower) such that punishers cannot improve their payoffs relative to those of targets. If the hypothesis that punishment sometimes represents aggressive competition for status is correct, then we would expect a marked decrease in antisocial punishment when punishers cannot improve their payoff relative to that of the target. It would also be interesting in any follow up to obtain self-reports from subjects on the emotions they feel prior to making their punishment decision. While some studies have suggested that punishment might often be preceded by negative emotions, such as anger or disgust (e.g. [3]), this might not necessarily be the case when punishment is motivated by a competitive drive to elevate relative status and is used indiscriminately against prosocial and antisocial partners.

While the data from the India-based subjects provides some support for the idea that punishment is proximately driven by competitive motives, the data from the US-based subjects suggest that revenge based motives cannot easily be ruled out. A similar conclusion was also reached by B&R, where it was shown that individuals would invest in 'inefficient' punishment (fee to fine: 1:1) if this was the only option available but that, when given access to an 'efficient' punishment option (fee to fine: 1:3), typically invested the amount that created equal outcomes for the punisher and the target [21]. Investment in inefficient punishment supports the idea that punishment is motivated by a desire for revenge rather than by competitive motives to equalize or increase payoffs relative to the target (since, by definition, inefficient punishment cannot have any bearing on relative payoffs of punisher and target). Nevertheless, the preference to equalize outcomes—when possible—supports the idea that punishment is motivated by egalitarian preferences and is therefore more likely to serve a fitness-leveling function. If the assumption that motives underpinning decisions can lend some insight into the likely evolved function of the behavior is correct, then—based on the previous data and the data collected for the current study—we suggest that punishment might in fact serve both a deterrent function (motivated primarily by revenge) and a fitness-leveling (or improving) function (motivated by competitive desire; which may be increased when punisher experiences disadvantageous inequality). The relative importance of the two functions (and associated motives) might be expected to vary according to context. Based on the current data, it appears that country of origin is likely to play an important role in determining the relative importance of the two functions (and underlying motives) of punishment, although it is not yet clear which of the many factors that vary across these two societies might be causal. Moreover, whether among-country variation in punishment patterns is greater than that which is observed within countries remains an open avenue for investigation.

A common concern with studies conducted using MTurk is that the results are obtained using small stakes compared to those that are used in more traditional laboratory settings, and may not therefore be representative. In general, however, these small stakes do not seem to meaningfully affect behaviour (at least compared to what is observed in the lab (see [26, 53, 54]). Nevertheless, Indian-based players do treat larger stakes (i.e. $10) differently to US-based players in a Dictator Game [26]. Reassuringly, in the range of stake sizes we used in the current study ($0.70-$1.10), Raihani et al. [26] reported no meaningful differences in Dictator Game sharing between US-based and India-based players. More importantly, even if there were differences in how players from India and the US, respectively, perceived the stakes we used in the current study, it is not clear what the predictions would be with respect to punishment decisions. One might predict either outcome: Indian players could be less inclined to punish, since punishing involves sacrificing a greater perceived sum; or more inclined to punish, since the stolen amount is perceived as more meaningful. It also isn't immediately obvious how different perceptions of value attached to the stakes would predict variation in punishment across treatments (where stakes were constant), as we observed. In short, although we don't dispute that $1 means something different to a US-based player than to an India-based player, it is not clear how or why this would have produced the pattern of results observed here.

We also acknowledge the possibility that the patterns of results could reflect differences in understanding the strategic incentives of the game, rather than differences in social preferences (c.f. [55]). Though possible, we believe this explanation is unlikely to account for the different patterns of punishment across the US and India-based players. First, all players were required to correctly answer three comprehension questions in order to be eligible to participate (S1 Appendix). Moreover, all participants saw images depicting their own and the partner's payoffs at the start of the game, the payoffs at the end of stage 1 (when P2 either stole or did not steal) and the payoffs that would ensue to them and to P2 if they decided to punish P2. This level of detail is in line with the 'enhanced information' condition described in [55] which enhanced participants' understanding of the strategic incentives of the game. Alternatively, broad-scale differences in propensity to punish could be due to fundamental differences between the individuals who make up the India and US sample. It is not yet clear what these differences might be, nor why they should render one category of individuals more punitive (and more prone to antisocial punishment) than the other but this possibility should nevertheless be considered.

To conclude, the discrepancies that we observed across two similar studies concerning the importance of revenge as a proximate motive underpinning punishment decisions do not seem to reflect minor methodological details or, as was even more of a concern, the general unreliability or noisiness of data collected online. Instead, we suggest that the observed differences reflect different demographic sampling and the fact that country of origin was not accounted for in the earlier R&M [19] study. Re-analysis of the R&M data [19], controlling for country or origin, reveals similar patterns as reported here [S1 Fig, S5 Appendix]. Moreover, comparing the data from our US-based players in this study with the comparable data obtained by B&R [21] reveals remarkable consistency in the overall patterns (and, in fact, others have also reported similar levels of consistency across studies conducted using MTurk, [54]). There is much more to be done in understanding what motivates punishment decisions and, even more importantly, why these motivations vary across contexts.

## Supporting Information

S1 Appendix Instructions given to subjects. (DOC)

S2 Appendix Information about the use of MTurk to recruit participants. (DOC)

S3 Appendix R code used to fit models. (DOC)

S4 Appendix Data for analysis. (CSV)

S5 Appendix Reanalysis of data from R&M. (DOC)

S6 Appendix Calculating norms of civic cooperation. (DOC)

S1 Fig Proportion of players from India and USA who punished P2 according to outcome, using data from R&M [19]. (DOC)

S1 Table Age, gender and country of origin for all individuals allocated to the role of P1. (DOC)

S2 Table Demographic information for India-based and US-based individuals allocated to the role of P1. (DOC)

S3 Table Explanatory terms included in top models for dependent variable 'P1 punished P2. (DOC)
