# Supplementary material for: Exploring the Motivations for Punishment: Framing and Country-Level Effects
Source: PLoS One. 2016 Aug 3;11(8):e0159769. doi: 10.1371/journal.pone.0159769 (PMC4972317; doi:10.1371/journal.pone.0159769)
Supplement: S1 Appendix — (DOC) [file pone.0159769.s001.doc]

**S1 Appendix. Instructions given to subjects**

Having completed the demographic questions, subjects were redirected to an external survey website ([https://opinio.ucl.ac.uk](https://opinio.ucl.ac.uk/)) to take part in the experiment. Below is a text transcription of the game instructions received by players, including comprehension questions. The example given is for a player 1 for the ‘replication’ treatment, scenario (A); however, the general procedure is similar for all treatments and punishment conditions

.

Screen 1: You are about to take part in an academic study which is run by Raihani Lab based at University College London. By continuing with the HIT you are consenting to allow Raihani Lab to use your responses in the study for academic purposes. All data are anonymous (your name or worker ID will not appear in any publication related to this study). Please tick 'I agree' if you agree to these conditions. If you do not wish to participate, or if you change your mind during the course of the study, please return to the Mechanical Turk Interface and click 'Return HIT'

Screen 2: Please enter your Worker ID. Your Worker ID is needed to ensure you get your bonus. Please DO NOT enter your email address or name in this box. If you don't know your Worker ID you can find it out by opening the following page in a new window: <https://www.mturk.com/mturk/dashboard>

Screen 3: You are playing a game with another worker. Your worker ID will not be revealed to the other player and you will not find out their worker ID. You are Player 1. You have been allocated $0.70 bonus. Player 2 has been allocated $0.10 bonus. This game has 2 stages. Stage 1: Player 2 can choose to take $0.20 from your bonus or to do nothing. Stage 2: You find out what Player 2 did in Stage 1 and can then choose one of the following options:

- [A] reduce Player 2's bonus by paying $0.10 to reduce Player 2's bonus by $0.30. - [B] do nothing (no cost to you or Player 2)

Screen 4: Before proceeding, please answer the following questions. Please answer carefully - you will not be able to proceed if you get an answer wrong. Reminder: You are Player 1. You have a $0.70 bonus. Player 2 has a $0.10 bonus.

1. If Player 2 takes $0.20 from your bonus in Stage 1 how much will you have at the start of Stage 2? (I will have $0.50 / I will have $0.70 / I will have $0.90)

2. If Player 2 takes $0.20 from your bonus in Stage 1, how much will Player 2 have at the start of Stage 2? (Player 2 will have $0.70 / Player 2 will have $0.30 / Player 2 will have $0.90)

3. How much do you have to pay to reduce Player 2's bonus in Stage 2? (Nothing - it is free / it costs me $0.50 to reduce Player 2's bonus / It costs me $0.10 to reduce Player 2's bonus)

Screen 5: Well done - you got all the questions right. Click 'continue' below to continue to the game. (Continue)

Screen 6: STAGE 1. Player 2 could choose to take $0.20 of your bonus or to do nothing. Player 2 took $0.20 of your bonus. The starting bonuses and current bonuses are shown below.


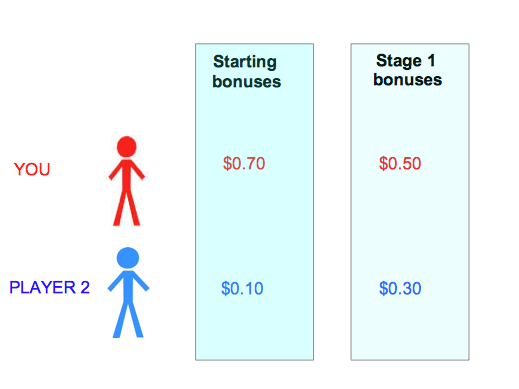


Screen 7: STAGE 2. You may now pay $0.10 to reduce Player 2's bonus by $0.30. The possible final bonuses for you and Player 2 are shown below. Do you want to reduce Player 2's bonus? (Yes - I want to reduce Player 2's bonus / No - I don't want to reduce Player 2's bonus )


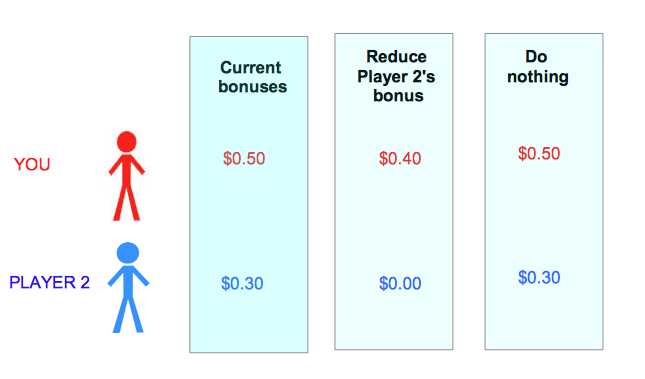


Screen 8: That's the end of the game. The mystery word is 'FISH'. Please return to the HIT and enter the word 'FISH' in the box before submitting your HIT. Thanks for playing!
