# Supplementary material for: Exploring the Motivations for Punishment: Framing and Country-Level Effects
Source: PLoS One. 2016 Aug 3;11(8):e0159769. doi: 10.1371/journal.pone.0159769 (PMC4972317; doi:10.1371/journal.pone.0159769)
Supplement: S2 Appendix — (DOC) [file pone.0159769.s002.doc]

**S2 Appendix. Mechanical Turk information**

Although several studies have demonstrated the validity of MTurk as a tool for collecting behavioural data (Horton et al. 2011; Rand 2012; Suri & Watts 2011), there are potential issues that experimenters have to be aware of when using this online platform for behavioural research. An advantage of using MTurk over typical  western, educated, industrialized, rich and democratic (WEIRD; Henrich et al. (2010) samples is that MTurk allows for recruitment of a more diverse demographic sample (Buhrmester et al. 2011) and for subjects from non-Western world cultures to also be included so that cross-cultural effects on behaviour can be explored (e.g. Raihani et al. 2013). Nevertheless, experimenters relinquish a degree of control over the experimental setting since they cannot be certain that subjects complete the task alone (although most report that they do; Chandler et al. 2014) and are not distracted by performing other tasks (e.g. instant messaging) simultaneously (Chandler et al. 2014). The use of attention checks, built in as comprehension questions, can be used to screen out subjects who either do not attend to or do not understand the nature of the task (Goodman et al. 2013). Although it is typical for subjects to earn smaller stakes on MTurk than when in the laboratory, hourly payment rates are usually comparable; and the effect of playing for a small stake has been shown to have no discernible effect on general patterns in standard economic games, at least among US-based subjects (e.g. Amir et al. 2012; Raihani et al. 2013). Perhaps most concerning is the finding that the subject base has become increasingly experienced with common behavioural experiments over time; and that performance in some tasks has been shown to vary with the level of experience (Rand et al. 2014). Specifically, in a study conducted on MTurk, Rand et al. (2014) showed that decisions made under time pressure varied systematically with subject experience. Despite this fact, other studies have shown subjects display remarkable consistency in responses, both across different games used to measure cooperative tendency conducted on MTurk and in self-reports of similar behavioural measures in real-life (Peysakhovich et al. 2014). On balance, therefore, we feel that so long as appropriate measures are taken to exclude subjects who do not pay attention to or do not understand the task, then MTurk should yield results that are comparable to those that could be obtained using other experimental settings.

Amir, O., Rand, D.G. & Gal, Y.K., 2012. Economic Games on the Internet: The Effect of 1 Stakes M. Perc, ed. *PLoS ONE*, 7(2), p.e31461.

Buhrmester, M., Kwang, T. & Gosling, S.D., 2011. Amazon’s Mechanical Turk: A New Source of Inexpensive, Yet High-Quality, Data? *Perspectives on Psychological Science*, 6(1), pp.3–5.

Chandler, J., Mueller, P. & Paolacci, G., 2014. Nonnaïveté among Amazon Mechanical Turk workers: consequences and solutions for behavioral researchers. *Behavior research methods*, 46(1), pp.112–30.

Goodman, J.K., Cryder, C.E. & Cheema, A., 2013. Data Collection in a Flat World: The Strengths and Weaknesses of Mechanical Turk Samples. *Journal of Behavioral Decision Making*, 26(3), pp.213–224.

Henrich, J., Heine, S.J. & Norenzayan, A., 2010. Most people are not WEIRD. *Nature*, 466(7302), p.29.

Herrmann, B., Thöni, C. & Gächter, S., 2008. Antisocial punishment across societies. *Science (New York, N.Y.)*, 319(5868), pp.1362–7.

Horton, J.J., Rand, D.G. & Zeckhauser, R.J., 2011. The online laboratory: conducting experiments in a real labor market. *Experimental Economics*, 14(3), pp.399–425.

Peysakhovich, A., Nowak, M.A. & Rand, D.G., 2014. Humans display a “cooperative phenotype” that is domain general and temporally stable. *Nature communications*, 5, p.4939.

Raihani, N.J., Mace, R. & Lamba, S., 2013. The effect of $1, $5 and $10 stakes in an online dictator game. Z. Barta, ed. *PloS one*, 8(8), p.e73131.

Rand, D.G. et al., 2014. Social heuristics shape intuitive cooperation. *Nature communications*, 5, p.3677.

Rand, D.G., 2012. The promise of Mechanical Turk: how online labor markets can help theorists run behavioral experiments. *Journal of theoretical biology*, 299(null), pp.172–9.

Suri, S. & Watts, D.J., 2011. Cooperation and contagion in web-based, networked public goods experiments. *PloS one*, 6(3), p.e16836.
