# Supplementary material for: Exploring the Motivations for Punishment: Framing and Country-Level Effects
Source: PLoS One. 2016 Aug 3;11(8):e0159769. doi: 10.1371/journal.pone.0159769 (PMC4972317; doi:10.1371/journal.pone.0159769)
Supplement: S3 Appendix — (DOC) [file pone.0159769.s003.doc]

**S3 Appendix. R code used to fit generalised linear model**

#Load data and packages

library(MuMIn)

library(arm)

data<-read.csv("data.csv", header=T)

#Model

global.model<-glm(Punished~Equality.ruined+Country*cbind(P2.stole.no.DI,P2.stole.DI),na.action=na.fail, family=binomial, data=data)

stdz.model<-standardize(global.model, standardize.y=FALSE)

model.set<-dredge(stdz.model, REML=FALSE)

top.models<-get.models(model.set, subset=delta<2)

m1<-model.avg(top.models, adjusted=FALSE, revised.var=TRUE)

summary(m1)

confint(m1)
