# Supplementary material for: Exploring the Motivations for Punishment: Framing and Country-Level Effects
Source: PLoS One. 2016 Aug 3;11(8):e0159769. doi: 10.1371/journal.pone.0159769 (PMC4972317; doi:10.1371/journal.pone.0159769)
Supplement: S5 Appendix — (DOC) [file pone.0159769.s005.doc]

**S5 Appendix. Reanalysis of data from R&M**

We wanted to explore whether the cross-cultural differences in the propensity to punish found in the current study were consistent with the R&M study. In the R&M study the vast majority (81 %) of subjects allocated to the role of P1 were recruited were from the US or India. In the R&M study there were more India-based P1s (n = 130) than US-based P1s (n = 97), whereas in the current study, we had more US-based P1s (n = 962) than India-based P1s (n = 176). Although sample sizes were too small to formally test for cross-cultural differences in the R&M data, descriptive statistics support the patterns found in the current study. Subjects from India were more likely than US-based subjects to punish a non-stealing partner (proportion India-based P1 punishing non-stealing P2 = 0.16 (0.08, 0.28); US-based P1 punishing non-stealing P2 = 0.03 (0.00, 0.12); Figure S1). So long as stealing did not result in disadvantageous inequality, India-based subjects did not punish a stealing partner more than a non-stealing partner. However, when stealing did result in disadvantageous inequality, India-based subjects were more likely to punish the partner (proportion India-based subjects P1 punishing stealing P2 when stealing did not result in disadvantageous inequity = 0.20 (0.11, 0.33); when stealing did result in disadvantageous inequity = 0.36 (0.20, 0.57); Figure S1b). The patterns for US-based subjects were different: even when stealing did not result in disadvantageous inequality, subjects were more likely to punish stealing than non-stealing partners (Figure S1a). Nevertheless, US-based subjects were also sensitive to inequality and punished stealing partners even more when stealing resulted in disadvantageous inequality (proportion US-based P1 punishing stealing P2 when stealing did not result in disadvantageous inequity = 0.1 (0.03, 0.26); when stealing did result in disadvantageous inequity = 0.67 (0.35, 0.88); Figure S1a).
