# Supplementary material for: Exploring the Motivations for Punishment: Framing and Country-Level Effects
Source: PLoS One. 2016 Aug 3;11(8):e0159769. doi: 10.1371/journal.pone.0159769 (PMC4972317; doi:10.1371/journal.pone.0159769)
Supplement: S6 Appendix — (DOC) [file pone.0159769.s006.doc]

**S6 Appendix. Calculating norms of civic cooperation**

We followed the methodology of Herrmann et al. (2008) to calculate the norms of civic cooperation for India relative to the USA from the period 2010 - 2014. We used data from the World Values Survey website (www.worldvalues.survey.org) and assessed the answers to the three questions previously analysed by Herrmann et al. 2008 to calculate the norms of civic cooperation for each country. These three questions asked participants to answer to what extent (on a scale of 1 - 10, with 1 being never justifiable and 10 being always justifiable) the following actions could be justified: claiming government benefits to which you are not entitled (question V198); cheating on taxes if you have a chance (V201); and avoiding a fare on public transport (V199). As in Herrmann et al. (2008) we rescaled the answers such that a value of 1 would indicate weak civic norms and a value of 10 would indicate strong civic norms. In the Herrmann et al. (2008) study, the authors typically used data collected between 1999-2004; in this analysis we used the data collected from 2010-2014. Our social capital variables for norms of civic cooperation for India and the USA, respectively, were 6.84 and 8.73. Previously Herrmann et al. (2008) reported a value of 8.65 for the USA (and a global range of 6.75 - 9.81). Thus, by this metric, India appears to have extremely weak civic norms, which has been associated with increased prevalence of antisocial punishment (Herrmann et al. 2008).
