# Supplementary material for: Exploring the Motivations for Punishment: Framing and Country-Level Effects
Source: PLoS One. 2016 Aug 3;11(8):e0159769. doi: 10.1371/journal.pone.0159769 (PMC4972317; doi:10.1371/journal.pone.0159769)
Supplement: S1 Fig — (DOC) [file pone.0159769.s007.doc]

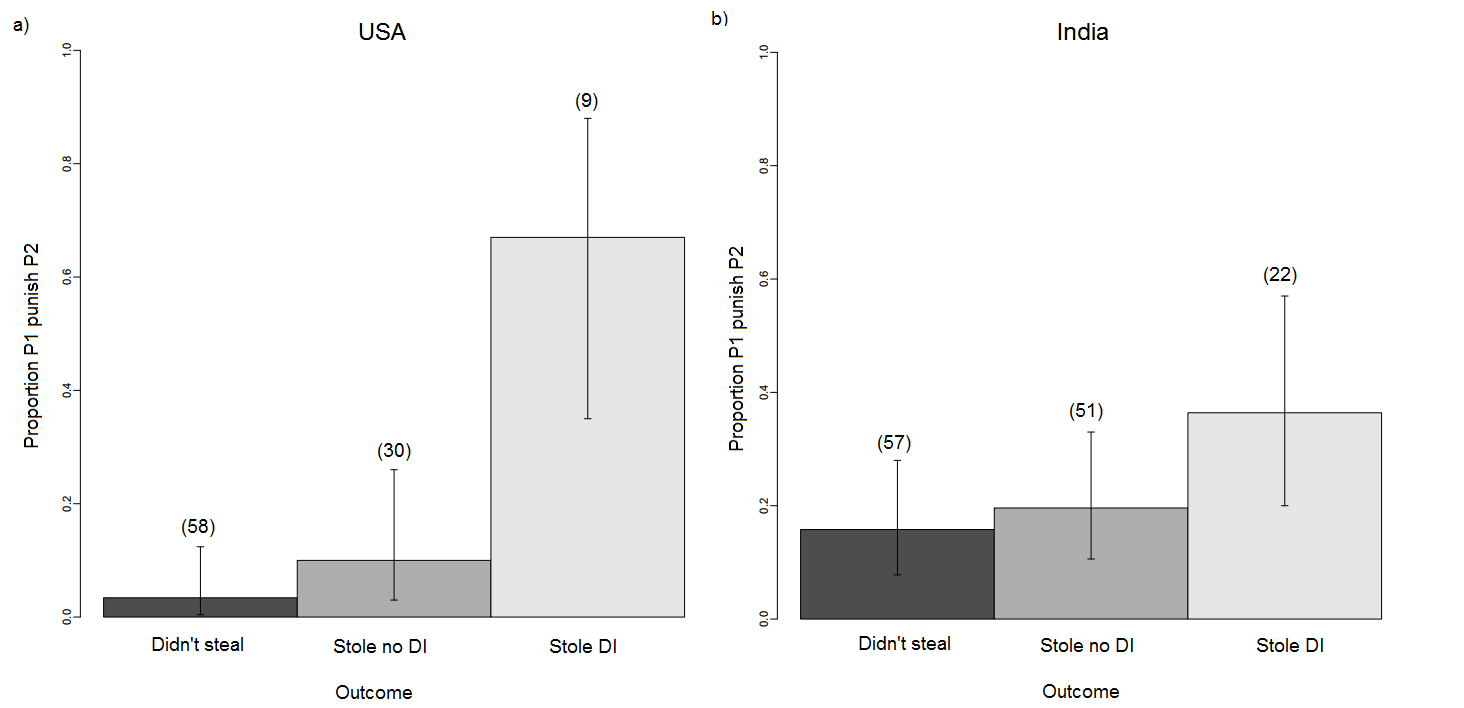


**Figure S1** The proportion of P1 who punished P2 when P2 didn’t steal (‘Didn’t steal’), P2 stole but the stealing did not result in disadvantageous inequality (‘Stole no DI’) or P2 stole and the outcome was disadvantageous inequality for P1 (‘Stole DI’) in data from the R&M study. Data are shown for players based in **a)** the USA and **b)** India. Error bars show the 95 % confidence intervals. Sample sizes for each condition are indicated in parentheses. Plots are generated from raw data.
