# Supplementary material for: Exploring the Motivations for Punishment: Framing and Country-Level Effects
Source: PLoS One. 2016 Aug 3;11(8):e0159769. doi: 10.1371/journal.pone.0159769 (PMC4972317; doi:10.1371/journal.pone.0159769)
Supplement: S1 Table — (DOC) [file pone.0159769.s008.doc]

**Table S1.** Age, gender and country of origin for all subjects allocated to role of player 1.

| **Parameter** | **P1 demographic information** (n = 1195) |
| --- | --- |
| Age | Mean = 31 ± 0.3  Range = 18 – 88 |
| Gender (n) | Females = 493 (42.0 %)  Males = 680 (58.0 %)  Undisclosed = 22 |
| Country | Australia = 1  Belgium = 2  Bolivia = 1  Brazil = 1  Bulgaria = 1  Canada = 6  China = 3  Croatia = 2  Czech Republic = 1  Ethiopia = 1  Germany = 1  India = 176  Indonesia = 1  Italy = 1  Lithuania = 1  Macedonia = 1  Panama = 1  Peru = 1  Philippines = 3  Poland = 1  Romania = 2  Russia = 1  Serbia = 2  Thailand = 1  UK = 1  USA = 962 |
