# Supplementary material for: Exploring the Motivations for Punishment: Framing and Country-Level Effects
Source: PLoS One. 2016 Aug 3;11(8):e0159769. doi: 10.1371/journal.pone.0159769 (PMC4972317; doi:10.1371/journal.pone.0159769)
Supplement: S2 Table — (DOC) [file pone.0159769.s009.doc]

**S2 Table.** Demographic information for US-based and India-based subjects allocated to role of player 1.

| **Parameter** | **India** (n = 176) | **USA** (n = 962) |
| --- | --- | --- |
| Age | Mean = 32.4 ± 0.6  Range = 19-72 | Mean = 31 ± 0.3  Range = 18 - 88 |
| Gender | Females = 57 (33 %)  Males = 116 (67 %)  Undisclosed = 3 | Females = 420 (44 %)  Males = 535 (56 %)  Undisclosed = 7 |
| Education | School = 6 (4 %)  Primary degree = 112 (66 %)  Graduate degree = 53 (31 %)  Undisclosed = 5 | School = 99 (10 %)  Primary degree = 749 (79 %)  Graduate degree = 100 (11 %)  Undisclosed = 14 |
| Income | Less than $12,000 = 88 (43 %)  $12,000 - $24,999 = 80 (39 %)  $25,000 - $49,999 = 25 (12 %)  $50,000 - $99,999 = 11 (5 %)  More than $100,000 = 1 (0.4 %)  Undisclosed = 11 | Less than $12,000 = 94 (10 %)  $12,000 - $24,999 = 154 (17 %)  $25,000 - $49,999 = 291 (32 %)  $50,000 - $99,999 = 284 (31 %)  More than $100,000 = 92 (10 %)  Undisclosed = 47 |
