# Supplementary material for: Exploring the Motivations for Punishment: Framing and Country-Level Effects
Source: PLoS One. 2016 Aug 3;11(8):e0159769. doi: 10.1371/journal.pone.0159769 (PMC4972317; doi:10.1371/journal.pone.0159769)
Supplement: S3 Table — (DOC) [file pone.0159769.s010.doc]

**S3 Table.** Explanatory terms included in top models for dependent variable 'P1 punished P2.

| **Parameter** | **Estimate** | **Unconditional**  **SE** | **Confidence Interval** | **Relative Importance** |
| --- | --- | --- | --- | --- |
| Intercept | -2.75 | 0.20 | (-3.14, -2.36) |  |
| Country | -1.93 | 0.30 | (-2.52, -1.35) | 1.00 |
| Outcome |  |  |  | 1.00 |
| P2 didn’t steal | -2.10 | 0.42 | (-2.93, -1.27) |  |
| P2 stole DI | 1.21 | 0.24 | (0.74, 1.67) |  |
| Outcome x Country |  |  |  | 1.00 |
| P2 stole no DI | -2.40 | 0.67 | (-3.71, -1.09) |  |
| P2 stole DI | -0.30 | 0.55 | (-1.37, 0.77) |  |
| Equality ruined | -0.05 | 0.17 | (-0.69, 0.35) | 0.31 |

**Table S3.** Estimates, unconditional standard errors, confidence intervals and relative importance for parameters included in the top models. All input variables were centred by subtracting the mean (Schielzeth 2010). Standard errors are unconditional, meaning that they incorporate model selection uncertainty. Outcome is a 3-level categorical variable ‘P2 didn’t steal’ = player 2 did not steal; ‘P2 stole no DI’ = player 2 stole but this did not result in disadvantageous inequality for P1; and ‘P2 stole DI’ = player 2 stole and this resulted in in disadvantageous inequality for P1. For outcome, P2 stole no DI' is the reference level.
